# For the Worlds Fair

**Published:** 1892-12

**Authors:** 


### For the World's Fair.

The question soon will be, and in the minds of many even now is, how best to get there. The inquiry will be answered somewhat with respect to the starting point. Taking Cincinnati as one of the points from which there will be an immense travel during the time of the Exposition, the Monon Route via the C. H. & D. R. R. presents unsurpassed facilities. It is the most direct route, its equipment is of the best, its management has no superior, the train service is unexceptionable. Nowhere will more gentlemanly and accommodating attendants be found than on the trains of this road.

